# Computer-Assisted Surgery Enables Beginner Surgeons, Under Expert Guidance, to Achieve Long-Term Clinical Results not Inferior to Those of a Skilled Surgeon in Knee Arthroplasty

**DOI:** 10.1007/s43465-022-00666-9

**Published:** 2022-06-22

**Authors:** Ricardo Larrainzar-Garijo, Elisa M. Molanes-López, Miguel Cañones-Martín, David Murillo-Vizuete, Natalia Valencia-Santos, Raul Garcia-Bogalo, Fernando Corella-Montoya

**Affiliations:** 1 Orthopedic and Trauma Department, Hospital Universitario Infanta Leonor, C/ Gran Via Este 80, 28031 Madrid, Spain; 2 Departamento Cirugía, Facultad de Medicina, Universidad Complutense Madrid, Madrid, Spain; 3 Unidad Departamental de BioestadísticaDepartamento de Estadística e Investigación Operativa, Facultad de Medicina, Universidad Complutense Madrid, Madrid, Spain

**Keywords:** Knee prosthesis, Arthroplasty, Replacement, Knee, Computer-assisted, Kinematic, Femorotibial mechanical angle, Dynamic alignment

## Abstract

**Purpose:**

The purpose of this study is to determine whether the use of a surgical navigation system in total knee replacement (TKR) enables beginner and intermediate surgeons to achieve clinical PROM outcomes as good as those conducted by expert surgeons in the long term.

**Methods:**

We enrolled 100 consecutive patients whose total navigated knee arthroplasty (TKA) was performed in our institution from 2008 to 2010. According to the principal surgeon's surgical experience, the patients were divided into three groups: (1) beginner surgeons, with no more than 30 previous knee replacement performances, (2) intermediate surgeons, with more than 30 but not more than 300, and (3) expert surgeons, with more than 300 knee replacements. Demographic data collected on the cohort included gender, laterality, age, and body mass index (BMI). The outcome measures assessed were Forgotten Joint Score (FJS), implant positioning, limb alignment, and prosthesis survival rate. A margin of equivalence of ± 18.5 points in the FJS scale was prespecified in terms of the minimal clinically important difference (MCID) to compare the FJS results obtained in the long period between the groups of interest.

**Results:**

The mean follow-up was 11.10 ± 0.78, 10.86 ± 0.66, and 11.30 ± 0.74 years, respectively, for each of the groups. The long-term FJS mean score was 80.86 ± 21.88, 81.36 ± 23.87, and 90.48 ± 14.65 for each group. The statistical analysis proved noninferiority and equivalence in terms of the FJS results reported in the long term by patients in Groups 1 or 2 compared to those in Group 3. More specifically, it has been proved that the mean difference between groups is within the interval of equivalence defined in terms of the MCID. The overall prostheses survival rate was 93.7%.

**Conclusion:**

Navigated assisted TKA, under expert guidance, can be as effective when performed by beginner or intermediate surgeons as performed by senior surgeons regarding the accuracy of implant positioning, limb alignment, and long-term clinical outcome.

## Introduction

In any human activity requiring learning, repetition improves results, especially in manual activities. Surgery is a clear example of this situation. Therefore, we assume that a surgical learning curve is always present and that surgical expertise can only be achieved after many years of clinical practice [1–4].

Computer-assisted navigated knee replacement provides surgeons with quantitative measurement tools for real-time assessing lower limb alignment and kinematics [5, 6]. It is a powerful instrument for intraoperatively supporting and guiding the surgeon in the adequate postoperative soft tissue balance of the knee [7–10].

Patient-reported outcome measures (PROMs) are standardized, validated questionnaires completed by patients to measure their perception of their functional well-being and health status. No single instrument has established itself as the 'gold standard' for measuring patient status. Each tool measures different dimensions of health, uses different scoring levels, and references different periods [11]. The Forgotten Joint Scores (FJS) comprise measures for assessing joint-specific patient-reported outcomes [12]. These PRO questionnaires focus on patients' awareness of a specific joint in everyday life and pick up subtle differences between patients and follow-up time points.

This study aims to determine if the use of a surgical navigation system enables beginner and intermediate surgeons to achieve long-term clinical PROM outcomes and postoperative implant positioning and limb alignment as good as those performed by an expert surgeon. We conducted a retrospective cohort study design based on a consecutive case series focused on the alternative hypothesis that the long-term FJS achieved by surgeons with less surgical experience is noninferior by a prespecified amount conducted by a skilled surgeon in navigated assisted total knee replacement. More specifically, the null hypothesis of inferiority specifies that the FJS between a less experienced surgeon and a skilled one is worse by at least a prespecified acceptable margin of 18.5 points, corresponding to the average of the smallest and largest minimal clinically important difference (MCID) estimates given by Ingelsrud et al. [13].

The main objective is to assess the long-term FJS result by comparing three surgeon's knowledge outlines: (1) no more than 30 previous knee replacement performances, (2) between 31 and 300, and (3) more than 300 knee replacements. Secondary objectives are to assess the postoperative Hip Knee Ankle Angle, implant position, and survival rate between the groups.

## Materials and Methods

**Fig. 1:**
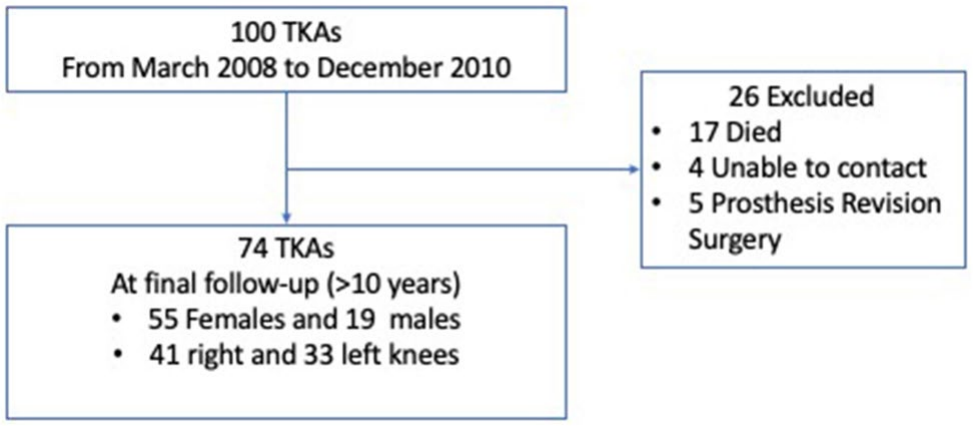
Patient flow diagram. *TKA* total knee arthroplasty

We enrolled in this study 100 consecutive patients in which navigated total knee arthroplasty (TKA) was performed in our institution from 2008 to 2010. Seventeen patients had died during the follow-up period until 12/31/2020. The inclusion criteria were patients with primary osteoarthritic knee joints and receiving a posterior stabilized total knee replacement (Columbus, BBraun Aesculap) due to substantial pain and loss of functionality with any degree of deformity. Of the 83 living patients, five were excluded due they had prostheses revision surgery. We could not contact four other patients by phone or mail, resulting in a final sample of 74 patients (Fig. 1).

All patients provided written informed consent, and the Hospital Committee for Medical and Health Research Ethics approved the study. (Hospital General Universitario Gregorio Marañón. Madrid. Spain. Protocol 1-04. V-02). All procedures performed followed the 1964 Declaration of Helsinki and its later amendments.

It is estimated that prosthetic knee surgeons must perform about 30 procedures a year to maintain their skills [14]. We, therefore, grouped surgeons into those who had not even reached the first thirty prostheses, those who had between 31 and 300, and more experienced surgeons with more than 300 implanted prostheses at the beginning of the study. According to the principal surgeon's surgical experience, the patients were divided into three groups: (1) no more than 30 previous knee replacement performances, (2) between 31 and 300, and (3) more than 300 knee replacements. A total of 14 surgeons were involved in the study.

Demographic data collected on the cohort included gender, laterality, age, and body mass index (BMI).

Baseline characteristics of the study population are reported in Table 1.

**Table 1:** Study population baseline

Patients characteristics	Group 1 *n* = 22	Group 2 *n* = 37	Group 3 *n* = 15	Global *N* = 74	*p* value
Female: *n* (%)	14 (63.6)	30 (81.1)	11 (73.3)	55 (74.3)	n.s.
Male: *n* (%)	8 (36.4)	7 (18.9)	4 (27.7)	19 (25.7)	n.s.
Right knee: *n* (%)	12 (54.5)	24 (64.9)	5 (33.3)	41 (55.4)	n.s.
Left knee: *n* (%)	10 (45.5)	13 (25.1)	10 (66.7)	33 (44.6)	n.s.
Age^a^ (years): mean ± SD	69.91 ± 8.67	72.00 ± 4.80	69.40 ± 7.48	70.85 ± 6.71	n.s.
BMI (kg/m^2^): mean ± SD	31.64 ± 3.59	33.17 ± 4.59	34.39 ± 5.40	32.96 ± 4.54	n.s.

*n.s.* no significant

^a^Patient age at the moment of the knee replacement

### Surgical Technique

The surgical procedure was performed following a navigated gap-balancing technique (Columbus PS. Orthopilot version 4.2; Braun Aesculap, Tuttlingen, Germany) in a regular fashion [5, 9]. A distal femoral cut was planned at 90° sagittal and coronal plane to the hip center. The tibial cut was planned at 90° coronal and 2° posterior slope sagittal to the ankle center. Femoral and tibial components were all cemented, and no patella was replaced.

### Outcome Measures

#### Implant Positioning and Limb Alignment

The navigation system assessed the coronal and sagittal HKAA (Hip Knee Ankle Angle) at the surgical procedure’s beginning and end once the cementation process was completed and the tourniquet was deflated. The joint orientation angles in the frontal and sagittal planes were evaluated according to Paley [15].

Femoral and tibial component position, joint line height, and gaps at 0° and 90° were calculated after all bone cuts were done. The navigation system referenced to the preoperative posterior condyle axis measured the final femoral implant rotation.

### Knee Balancing

To allow comparison of final knee balance between the three groups of surgeons, the authors classified the relationship between the medial and lateral gap, both in extension and flexion.

According to the most restrictive criterion, a knee is adequately balanced when there is no more than 2 mm difference in any of the four gap measurements (Flexion Medial Gap, Extension Medial Gap, Flexion Lateral Gap, Extension Lateral Gap). Two less restrictive criteria were defined similarly, considering three and four millimeters difference. Any value greater than five was regarded as inadequate knee balance.

### Forgotten Joint Score

All eligible patients were asked to complete the FJS questionnaire at the end of the follow-up period. The FJS was assessed by the same author (NVS). The FJS is used to evaluate patients' ability to forget their artificial joints in daily life. It consists of 12 questions, and the score ranges from 0 to 100. The higher the score, the more favorable the outcome. The score was calculated under the original publication [12].

### Survival Rate

Prosthetic failure is defined as any clinical circumstance that removes the prosthesis, either due to aseptic loosening or prosthetic infection. All patients were evaluated until the end of the follow-up period:12/31/2020.

### Statistical Analysis

Traditional tests for differences were used to compare patients’ baseline characteristics, postoperative implant positioning, and limb alignment. For quantitative variables, one-way ANOVA was used under the assumption of Normality. For non-normally distributed data, the Kruskal–Wallis test was used instead. The premise of Normality was checked using the Shapiro–Wilk test.

For the primary quantitative outcome, one-sided tests were used to test for noninferiority between each group of less experienced surgeons and the group of more skilled surgeons. Due to the non-normality of the FJS values, these one-sided tests were based on a robust version of the two-sample Student *t* test that uses trimmed summary statistics, allowing for heterogeneity and deviations from Normality (the Yuen–Welch *t* test with a 5% trimming at both ends of the data). Given that there are two comparisons of interest (Group 1 versus Group 3 and Group 2 versus Group 3), Holm's sequential Bonferroni (HB) correction was used to control the family-wise error rate at the prespecified significance alpha level [16].

The HB method is a less conservative approach than the Bonferroni method that compares the *k*-ranked *p* value to the nominal significance level divided by (*n* − *k* + 1), wherein in this case, *n* = 2 (the number of comparisons of interest) and *k* = 1, 2.

The FJS-12 has a minimally clinically significant difference (MCID) of 14–23 points, as estimated by Ingelsrud et al. [13] Based on the average of these two most extreme MCID estimates, we define the interval of equivalence for the difference of FJS mean scores to be in the range from − 18.5 to 18.5 points; that is, the margin of noninferiority is given by delta = − 18.5. For the interval of equivalence previously defined and the nominal alpha level of 5%, 15 patients are required per group to prove the equivalence between groups of less experienced surgeons and the group of more skilled surgeons, with a statistical power of 80%. This sample size was calculated assuming an expected population standard deviation of 16.72 on the FJS-12 scale.

Finally, Kaplan–Meier curves were obtained to describe the survival of the prostheses, according to the principal surgeon's surgical experience.

The analyses were carried out using two statistical packages: SPPS version 25 and R version 4.0.4. A significance alpha level of 0.05 was set for all statistical tests.

## Results

### Limb Alignment

No statistically significant differences between the groups were demonstrated in the preoperative coronal and sagittal HKAA, making them comparable. There were no statistically significant differences in comparing postoperative coronal and sagittal HKAA between the groups.

Table 2 describes the preoperative and postoperative coronal and sagittal KHAA between the three groups globally.

**Table 2:** Comparison of preoperative and postoperative coronal and sagittal KHAA between the three different groups

Limb alignment	Group 1 *n* = 22	Group 2 *n* = 37	Group 3 *n* = 15	Global *N* = 74
Preoperative coronal HKAA	175.77 ± 5.98 (173.12; 178.43) 175.50 (172, 178)	174.62 ± 5.92 (172.65; 176.60) 175.00 (172, 176)	175.00 ± 5.96 (171.70; 178.30) 175.00 (171, 177.5)	175.04 ± 5.89 (173.68; 176.40) 175.00 (172, 178)
Preoperative sagittal HKAA	177.32 ± 7.11 (174.16; 180.47) 177.50 (176, 180)	178.27 ± 6.95 (175.95; 180.59) 180.00 (175, 182)	177.53 ± 6.46 (173.96; 181.11) 177.00 (173.5, 182)	177.84 ± 6.82 (176.26; 179.42) 178.00 (175, 182)
Postoperative coronal HKAA	180.32 ± 1.49 (179.66; 180.98) 180 (179, 182)	180.27 ± 1.47 (179.78; 180.76) 180 (179, 181)	179.93 ± 1.22 (179.26; 180.61) 180 (179, 181)	180.22 ± 1.42 (179.89; 180.54) 180 (179, 181)
Postoperative sagittal HKAA	181.95 ± 3.33 (180.48; 183.43) 181.5 (180, 185)	183.81 ± 2.93 (182.83; 184.79) 184 (182, 186)	183.93 ± 3.26 (182.13; 185.74) 183 (181.5, 186.5)	183.28 ± 3.20 (182.54; 184.02) 183 (181, 185)

Summary statistics are: mean ± SD, (95% CI) and median (Q1, Q3). There were no statistically significant differences in the comparison of postoperative coronal and sagittal HKAA between the groups

**Fig. 2:**
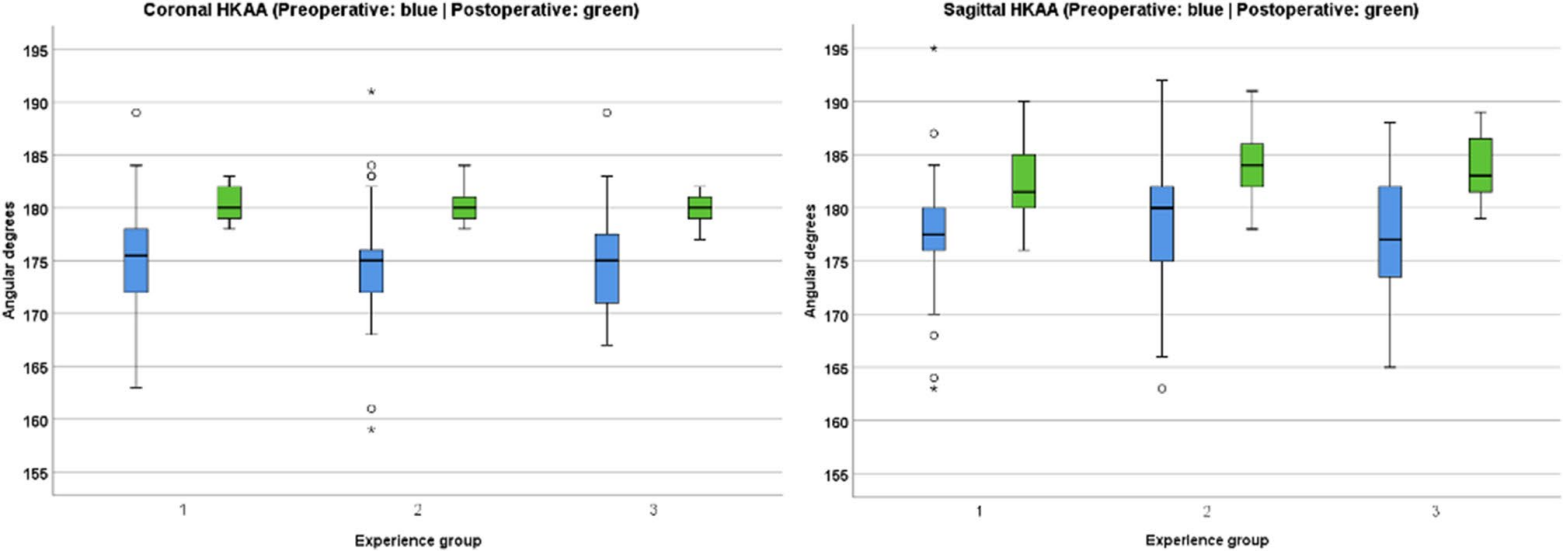
Coronal and sagittal alignment. The horizontal line in the box represents the median value. The height of the box is the interquartile range, Q1–Q3, i.e., where the central 50% of the most representative values are found. The vertical outbox lines represent the minimum and maximum of the non-outliers; when a value deviates from the top or bottom of the box more than 1.5 or 3 times the interquartile range, it is identified as an outlier or extreme outlier and expressed as a circle or a star

Figure 2 graphically expresses, through a boxplot, the homogeneity in the coronal and sagittal alignment (green color) between the different surgical experience groups. It can be seen that all the surgeons achieved close to neutral alignment in the coronal plane. At the same time, the HKAA was lightly recurvatum in the sagittal plane, especially between the most experienced surgeons, despite no statistically significant differences.

There was no outlier value in the coronal and sagittal postoperative alignment in any group.

### Implant Positioning

Only femoral component rotation showed statistically significant differences. The more experienced surgeons group provided more external rotation to the femoral component with a non-clinically relevant mean difference of 1°. Table 3 shows the implant position description according to the different surgical experience groups globally.

**Table 3:** Implant positioning descriptive evaluation between the three different groups

Variable	Group 1 *n* = 22	Group 2 *n* = 37	Group 3 *n* = 15	Global *N* = 74
Femur implant slope	89.91 ± 0.92 (89.50; 90.32) 90 (89, 90)	90.16 ± 0.90 (89.86; 90.46) 90 (90, 91)	90.40 ± 0.91 (89.90; 90.90) 90 (90, 91)	90.14 ± 0.91 (89.92; 90.35) 90 (90, 91)
Femoral implant rotation	1.45 ± 1.41 (0.83; 2.08) 2 (0, 3)	1.51 ± 1.39* (1.05; 1.98) 2 (1, 2)	2.47 ± 0.83* (2.00; 2.93) 3 (2, 3)	1.69 ± 1.34 (1.38; 2.00) 2 (1, 3)
Tibial implant slope	87.55 ± 1.47 (86.89; 88.20) 87 (87, 89)	87.65 ± 1.40 (87.18; 88.12) 88 (87, 88)	87.40 ± 1.24 (86.71; 88.09) 87 (86.5, 88)	87.57 ± 1.38 (87.25; 87.89) 88 (87, 89)
Joint line height	− 1.23 ± 2.31 (− 2.25; − 0.20) − 1 (− 2, 0)	− 0.08 ± 2.09 (− 0.78; 0.61) 0 (− 1, 1)	− 1.07 ± 3.03 (− 2.75; 0.61) − 1 (− 2.5, 1)	− 0.62 ± 2.40 (− 1.18; − 0.07) − 1 (− 2, 1)
Extension medial gap	1.86 ± 1.78 (1.07; 2.65) 2 (1, 2)	1.11 ± 1.58 (0.58; 1.63) 1 (0, 2)	1.93 ± 2.09 (0.78; 3.09) 2 (0.5, 2.5)	1.50 ± 1.77 (1.09; 1.91) 1 (0, 2)
Extension lateral gap	1.95 ± 1.91 (1.11; 2.80) 2 (0, 3)	2.05 ± 1.86 (1.44; 2.67) 2 (1, 3)	2.60 ± 1.92 (1.54; 3.66) 2 (1.5, 3.5)	2.14 ± 1.88 (1.70; 2.57) 2 (1, 3)
Flexion medial gap	3.91 ± 2.99 (2.58; 5.24) 3 (2, 5)	3.14 ± 2.10 (2.44; 3.83) 3 (2, 4)	3.67 ± 1.40 (2.89; 4.44) 4 (2, 5)	3.47 ± 2.29 (2.94; 4.00) 3 (2, 5)
Flexion lateral gap	3.27 ± 2.69 (2.08; 4.47) 3 (2, 4)	3.05 ± 1.70 (2.49; 3.62) 3 (2, 4)	3.20 ± 1.86 (2.17; 4.23) 3 (2, 4)	3.15 ± 2.05 (2.67; 3.62) 3 (2, 4)

These variables were obtained from the navigation system's final report provided by default. Summary statistics are: mean ± SD, (95% CI), and median (Q1, Q3)

*Only femoral component rotation showed statistically significant differences *p* < 0.05

### Long-Term Forgotten Joint Score

Seventy-four patients completed the FJS at the end of the follow-up period. FJS scale scores showed a non-normality distribution of the data. For a more robust statistical analysis of the FJS values, 5% trimmed summary statistics were considered (Table 4).

**Table 4:** FJS scores between the three different groups

FJS-12	Group 1 *n* = 22	Group 2 *n* = 37	Group 3 *n* = 15	Global *N* = 74
Mean ± SD	80.86 ± 21.88	81.36 ± 23.87	90.48 ± 14.65	83.06 ± 21.77
5% trimmed mean ± SD	83.61 ± 12.66	82.38 ± 20.55	90.48 ± 10.15	85.08 ± 16.72

Summary statistics are mean ± SD, and 5% trimmed mean ± SD

**Fig. 3:**
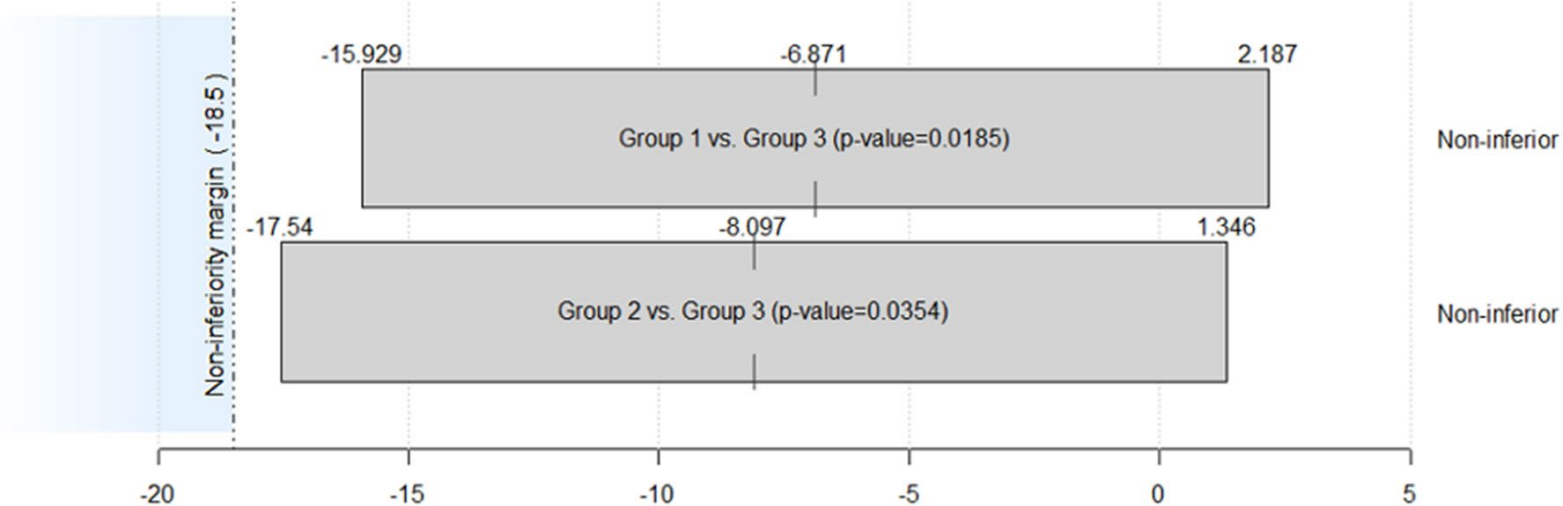
The noninferiority of the scores on the FJS scale graphically, considering Group 3 as a reference. Groups have been defined in “Material and Methods”. The alternative hypothesis (H1) states that the scores on the FJS scale cannot be worse than 18.5 points lower than in Group 3 (the group of skilled surgeons). According to the Minimal Clinically Important Difference, the pre-established margin of noninferiority is defined as the mean between the smallest and largest estimate of the MCID following Ingelsrud et al. [13]. Thus, any value located in the blue area (H0) represents clinical inferiority concerning Group 3

The statistical analysis proved noninferiority (and equivalence) for Groups 1 and 2 for Group 3, representing clinically that the beginner and intermediate surgeons achieved long-term clinical PROM results not inferior (and equivalent) to those of a skilled surgeon. As can be seen in Fig. 3, for both comparisons of interest, the corresponding 90% CIs for the mean difference (derived from the Yuen–Welch *t* test) are inside the interval of equivalence given by (− 18.5, 18.5), with first ranked *p* value = 0.0185 < 0.025 and second-ranked *p* value = 0.0354 < 0.05 (proving equivalence at a 5% significance level, based on the Holm–Bonferroni correction).

Note that the score on the FJS scale is a favorable or beneficial outcome and, consequently, the higher the values, the better.

**Fig. 4:**
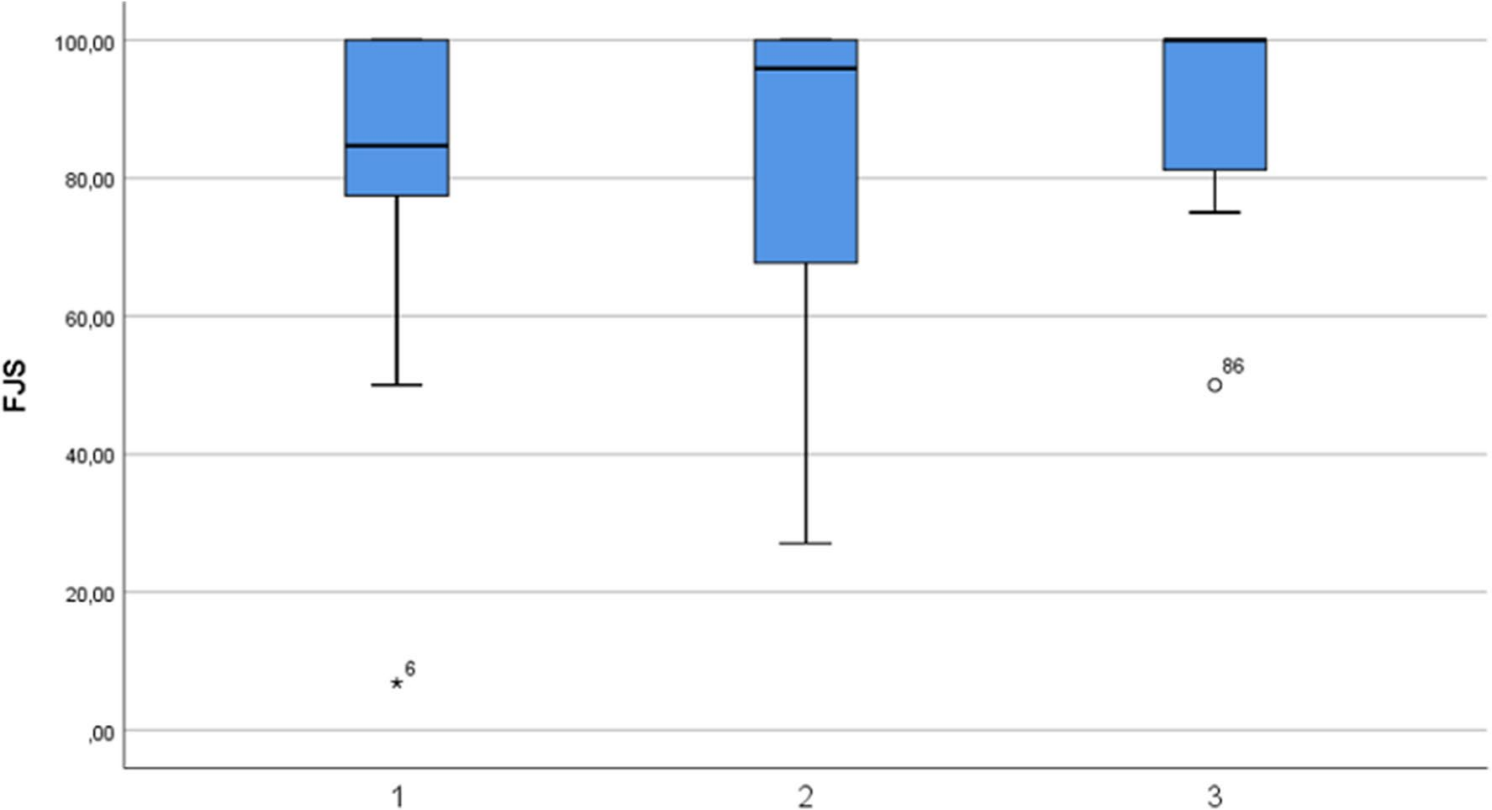
FJS. The horizontal line in the box represents the median value. The height of the box is the interquartile range, Q1–Q3, i.e., where the central 50% of the most representative matters are found. The vertical outbox lines represent the minimum and maximum of the non-outliers; when a value deviates from the top or bottom of the box more than 1.5 or 3 times the interquartile range, it is identified as an outlier extreme outlier and expressed as a circle or a star. Groups have been defined in “Material and Methods”. For example, group B has the greatest dispersion between the values in the FJS, but it is also the one with the most assigned patients. Note that the least experienced surgeons achieve the worst median FJS score

The most experienced surgeons tend to achieve better scores at FJS with less dispersion between values, representing a more consistent outcome. (Fig. 4).

### Survival Rate

Seventeen patients died before the long-term assessment. It was impossible to contact another four patients. None of these twenty-one patients required knee revision, according to clinical records.

The overall prostheses survival rate with a follow-up greater than ten years is 93.7%. Five patients required revision surgery related to aseptic loosening during the follow-up period. There were no diagnosed prosthetic infections.

The mean follow-up was 11.10 ± 0.78, 10.86 ± 0.66, and 11.30 ± 0.74 years, respectively.

**Fig. 5:**
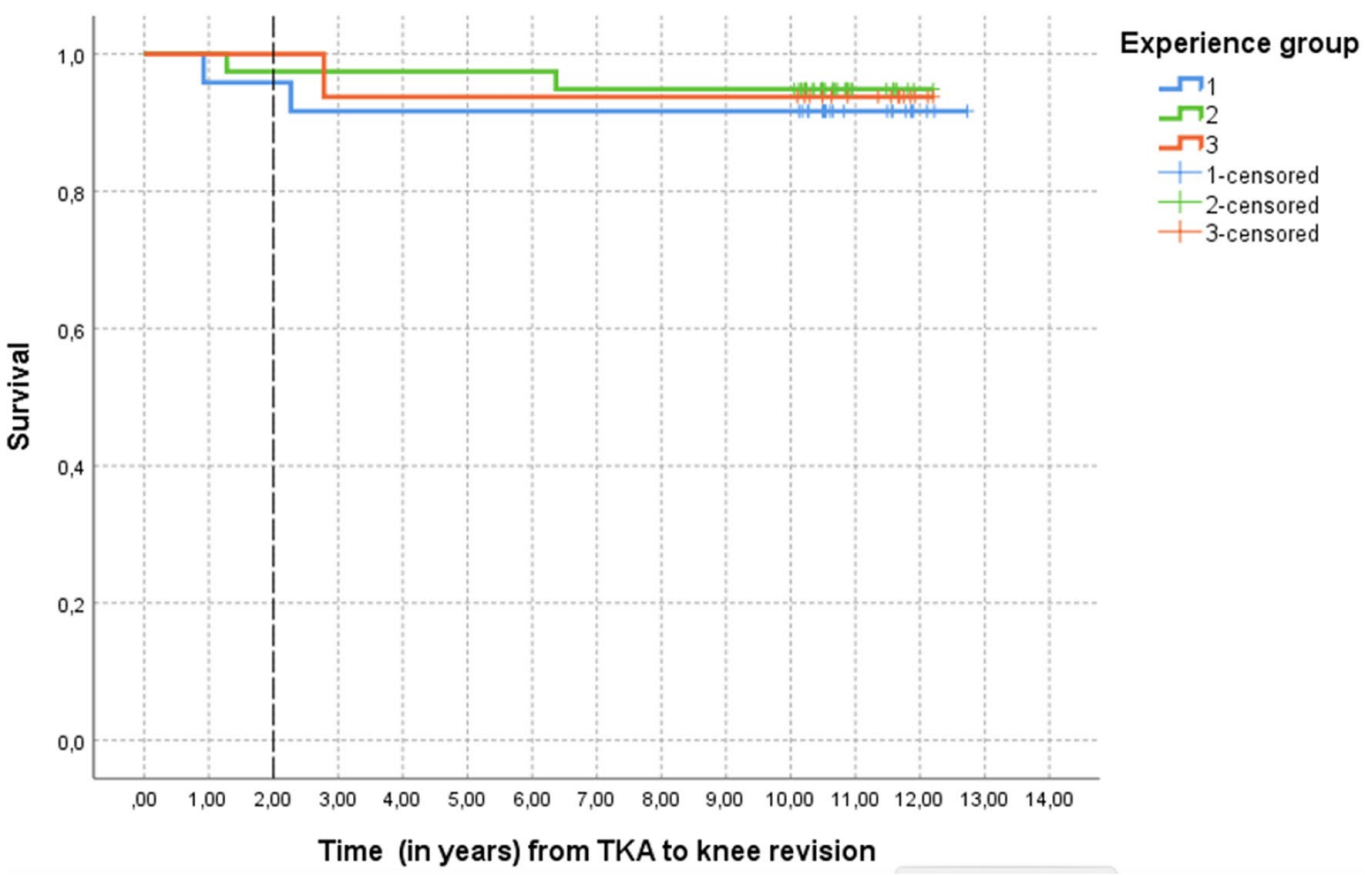
Kaplan–Meier graph showing prostheses survival. Groups have been defined in “Material and Methods”. Color code (1 = blue, 2 = green, 3 = red). Five patients required knee revision, two of them in the first two years and corresponding to groups 1 and 2

Figure 5 shows the Kaplan–Meier survival curves. The need for revision surgery occurs mainly in the first three years after surgery.

## Discussion

The most important finding of the present study is that long-term outcomes are equivalent between surgeons with different clinical experiences. The common denominator in all surgeries was using a surgical navigator to assist the surgeon during the procedure.

In 2008 our hospital was opened with a mix of surgeons with different previous experiences. All procedures are performed in our department, assisted by a navigation system. This unusual situation has allowed us to determine whether a surgical navigator can equalize results among surgeons. We measured the outcomes of interest in two different moments. We assessed the implant placement, alignment, and prosthetic stability at the end of the surgical procedure. In contrast, the long-term outcome was measured through a PROM assessment after more than ten years of follow-up.

There are many ways to measure clinical outcomes [17, 18], and any evaluation of the effectiveness of TKA depends on the definition of "successful". Previous studies have shown that 10–25% of patients are dissatisfied with the outcome of knee replacement at one to three years [19, 20]. In this situation, it is necessary to focus on quantifying the success of these procedures using patient-reported outcome measured (PROMS). The most popular PROMS assessing TKA outcomes are the WOMAC, Knee Injury and Osteoarthritis Outcome (KOOS) Score, and the Oxford Knee Score (OKS). Despite their many similarities, when using PROMs to assess TKA outcomes, the statistical significance (a *p* value) must be reported, and the clinical importance using the minimum clinically important difference (MCID) reported in the literature. The most crucial advantage of noninferiority and equivalence trials is that both designs allow comparison with currently existing, clinically accepted treatment, even if there is a ceiling effect [18].

The Forgotten joint score represents a valid and sensible PROM score with a low ceiling effect [12]. The low ceiling effect limits other scores such as WOMAC and OKS when detecting small clinical changes in patients who report good results.

Ingelsrud et al. reported an MCID of the FJS-12 in TKA between 14 and 23 points [13]. Defining the margin of equivalence as the average of these two values ((14 + 23)/2 = 18.5), we have proven equivalence of the beginner and intermediate surgeons concerning the expert group, with mean scores on the FJS scale of 80.86 ± 21.88, 81.36 ± 23.87, and 90.48 ± 14.65, respectively. In this sense, we believe our study demonstrates a similar outcome between beginners and experienced surgeons with a follow-up that exceeds ten years. The same observer (NVS) carried out all the patient interviews. The global results are slightly higher than those reported in the literature. This difference may be due to some positive bias. If this scale use bias were present, it would be uniform across the three groups. Our work does not want to compare the results obtained in our series with other published ones but rather to compare the surgeons in our study.

There were no statistically significant differences between the groups regarding the final alignment and the position of the femoral and tibial components. The overall result of a knee replacement depends on many factors, almost all related to the implant placement. The navigation system acts as a support tool, displaying real-time implant position and limb alignment, allowing the beginner surgeon to access consistent and relevant information through the procedure [5, 9, 10, 21, 22].

Although we have not found statistically significant differences between postoperative alignment, it should be noted that the sagittal HKAA is closer to neutral in the less experienced surgeon group.

There was no statistically significant correlation between implant alignment and position and the patient's subjective satisfaction measured on the FJS scale. This situation may indicate that both measures (objective clinical and subjective satisfaction) are complementary when evaluating the postoperative success of the prosthesis [18].

Good knee balancing is traditionally related to an excellent clinical outcome [23]. However, there is no direct correlation between a balanced prosthesis and an excellent clinical result [21, 24]. For objectivity, we used the principles of symmetry and congruence between the flexion and extension gaps to establish the comparison between the groups. It is remarkable that regardless of the measure, two, three, or four millimeters apart, an inexperienced surgeon, assisted by a navigation system, can achieve the same balance parameters as an experienced one, as shown in Fig. 3.

The overall prostheses survival rate with a follow-up greater than ten years is 94%. This value is slightly higher than that published in the registries [25] and may be based on the fact that, as mentioned, a surgical navigation system was used in all cases [26, 27]. As expected [20], most revision surgeries were indicated in the first three years.

The findings of this study will enable healthcare professionals to understand better the impact of implementing navigated assisted TKA on the surgical workflow, especially among less experienced surgeons or in those centers with a low volume of annual surgeries. Prosthetic knee surgery has changed in the last decade from a technique performed exclusively by experienced arthroplasty surgeons to almost a fundamental demand for any orthopedic surgeon. We must not forget that nearly all revision surgeries occur in the first two years after implantation and directly relate to the surgeon [20]. We have proven that relying on an external navigation system for intraoperative decision-making allows the surgeon to perform it as safely and efficiently as an experienced surgeon. Hospital managers should consider these findings, which would finance these systems, especially if the volume of prostheses per year is not very high.

There are several limitations to this study. First, there was no randomization between the allocation of cases and surgeons. The surgeries were performed consecutively and distributed according to the daily workload. We have conducted extensive statistical analyses to compare demographic values and prior alignment between patients. There are no statistically significant differences between them, affirming that the groups are comparable. Second, we have not considered the learning curve effect for navigation for each surgeon [14]. At least one experienced navigational surgeon in all the surgeries as part of the team. We sincerely believe that the presence of this surgeon in the team does not invalidate the results obtained since the role he played was secondary, leaving the leading surgeon to make decisions. We highlight this limitation through an express mention in the title and conclusion to provide the reader with a truthful judgment on the applicability of the study results. Recently it has been reported that Robotic-arm-assisted total knee arthroplasty has a learning curve of seven cases for integration into the surgical workflow but no learning curve effect for accuracy of implant positioning [28, 29]. It is more than possible that the same happens in navigation-assisted surgery since its data collection and surgery workflow have many similarities.

## Conclusion

Navigated assisted TKA, under expert guidance, can be as effective when performed by beginner or intermediate surgeons as performed by senior surgeons regarding the accuracy of implant positioning, limb alignment, and long-term clinical outcome.

## Data Availability

The datasets generated during and/or analyzed during the current study are available from the corresponding author on reasonable request.
